# Location, location, location: differential profiling of the nucleolar proteome of T-cells expressing HIV-1 Tat reveals the coordinated spatial control of distinct cellular networks

**DOI:** 10.1186/1742-4690-8-S2-P24

**Published:** 2011-10-03

**Authors:** Mohamed Ali Jarboui, William W Hall, Virginie W Gautier

**Affiliations:** 1 Centre for Research in Infectious Diseases, University College Dublin, Belfield, Dublin, Ireland

## Background

The trans-activator Tat protein is a viral regulatory protein essential for HIV-1 replication. Tat localises in both the nucleoplasm and the nucleolus. The nucleolus, a highly structured and dynamic membrane-less sub-nuclear compartment, is the site of rRNA and ribosome biogenesis and is involved in numerous cellular functions including transcriptional regulation, cell cycle control, RNA trafficking and viral infection. Importantly, transient nucleolar trafficking of both Tat and HIV-1 viral transcripts are critical in HIV-1 replication, however, the role(s) of the nucleolus in HIV-1 replication remains to be elucidated.

To better understand how the interaction of Tat with the nucleolar machinery contributes to HIV-1 pathogenesis, we investigated the quantitative changes in the composition of the nucleolar proteome of T-cells stably expressing HIV-1 Tat.

## Materials and methods

We employed an organellar proteomic approach using mass spectrometry coupled with Stable Isotope Labelling in Cell culture (SILAC). Identification, quantification and analysis of the purified nucleolar proteins was performed using MaxQuant, combined with Perseus, and cytoscape for pathway analysis and network reconstruction.

## Results

### Tat causes significant changes in the composition of the nucleolar proteome

We quantified over 450 proteins, including 45 proteins showing significant changes in abundance in the T-cell nucleolus upon HIV-1 Tat expression. Importantly, the majority of proteins exhibited an increase rather than a decrease in abundance. To validate our SILAC-based quantitative analysis, we performed WB analysis of the nucleolar fractions, and observed the concordance in the trend of fold change for the 15 selected proteins despite differences in the quantitative ranges.

### Tat nucleolar networking

Numerous proteins exhibiting a fold change were well characterised Tat ínteractors and/or known to be critical for HIV-1 replication. Pathway analysis and network reconstruction revealed that Tat expression specifically resulted in the nucleolar enrichment of proteins collectively participating in signaling pathways or macromolecular complexes associated with ribosomal biogenesis, intracellular trafficking, protein turn over, stress response, genome integrity and/or cell cycle control.

### Tat crowd control

WB analysis of the different subcellular fractions indicated that the pattern of Tat-mediated spatial redistribution of individual protein varied. Indeed, we could observe proteins shifting directly from the cytoplasm to the nucleolus or from the cytoplasm to the nucleus including the nucleolus, or from the nucleoplasm to the nucleolus. These observations suggest that the targeting to the nucleolus by Tat may involved distinct mechanisms and may depend on properties inherent for each protein. We also observed cases where Tat expression modulated the overall expression of specific proteins.

## Conclusions

Our proteomic analysis revealed that Tat controls the segregation and compartimentalisation of well-defined subsets of cellular proteins to the nucleolus. We discuss here how these proteins collectively participate in interconnected networks converging to adapt the nucleolus dynamic activities and may contribute to create a cellular environment favorable to HIV-1 replication.

